# Bioinspired Soft Robots Based on the Moisture‐Responsive Graphene Oxide

**DOI:** 10.1002/advs.202002464

**Published:** 2021-03-03

**Authors:** Yu‐Qing Liu, Zhao‐Di Chen, Dong‐Dong Han, Jiang‐Wei Mao, Jia‐Nan Ma, Yong‐Lai Zhang, Hong‐Bo Sun

**Affiliations:** ^1^ State Key Laboratory of Integrated Optoelectronics College of Electronic Science and Engineering Jilin University 2699 Qianjin Street Changchun 130012 China; ^2^ State Key Laboratory of Precision Measurement Technology and Instruments Department of Precision Instrument Tsinghua University Haidian District Beijing 100084 China

**Keywords:** actuators, bionics, graphene oxide, moisture responsiveness, soft robots, water molecules

## Abstract

Graphene oxide (GO), which has many oxygen functional groups, is a promising candidate for use in moisture‐responsive sensors and actuators due to the strong water–GO interaction and the ultrafast transport of water molecules within the stacked GO sheets. In the last 5 years, moisture‐responsive actuators based on GO have shown distinct advantages over other stimuli‐responsive materials and devices. Particularly, inspired by nature organisms, various moisture‐enabled soft robots have been successfully developed via rational assembly of the GO‐based actuators. Herein, the milestones in the development of moisture‐responsive soft robots based on GO are summarized. In addition, the working mechanisms, design principles, current achievement, and prospects are also comprehensively reviewed. In particular, the GO‐based soft robots are at the forefront of the advancement of automatable smart devices.

## Graphical abstract

In recent years, moisture‐responsive actuators based on graphene oxide (GO) have revealed a series of distinct advantages over other stimuli‐responsive materials and devices. In this research news, the milestones in moisture‐responsive soft robots based on the GO, including working mechanisms, design principles, current achievement, and prospects, are comprehensively reviewed.

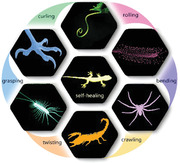

## 1 Introduction

Stimuli‐responsive actuators (SRAs) that directly convert environmental signals to mechanical work have been considered as key components in the development of micro‐electro‐mechanical‐systems (MEMS) and robotic devices.^[^ ^1^, ^2^, ^3^, ^4^, ^5^ ^]^ Through their sensitive response to environmental stimuli (e.g., temperature, magnetic field, electric field, humidity, and chemical reagents),^[^ ^6^, ^7^, ^8^, ^9^, ^10^ ^]^ SRAs enable rapid and dynamic actuation in a reversible and predictable manner. In particular, SRAs that can be manipulated via environmental changes without coupling with additional energy supply systems are emerging as the preferred driving components for next‐generation intelligent products.^[^ ^11^, ^12^, ^13^, ^14^, ^15^ ^]^ Previous studies have shown that rapid progress has been made in bio‐inspired functional materials that utilize switchable superwettability, smart interfacial materials, and intelligent structural color.^[^ ^16^, ^17^, ^18^ ^]^ Recently, natural organisms, which exhibit reversible structural deformation, have inspired the rational design of artificial SRAs for robotic applications. For instance, the opening of seed pods, burial of seed awns, winding of plant tendrils, and snapping of Venus flytrap are all due to moisture actuation.^[^ ^19^, ^20^, ^21^, ^22^, ^23^, ^24^ ^]^ The intrinsic mechanisms responsible for these structural deformations can be attributed to water absorption, dehydration of cells, or changes in internal hydrostatic pressure. To date, research studies on the morphing or motion in nature have provided key paradigms for natural shape‐changing, which has inspired the innovative development of their synthetic equivalents.^[^ ^25^, ^26^, ^27^, ^28^ ^]^ Consequently, soft materials, which have a special interaction with water molecules, have been widely employed in designing moisture‐responsive actuators in a biomimetic manner.

Graphene oxide (GO), a derivative of graphene, that possesses many hydrophilic oxygen‐containing‐groups (OCGs) has emerged as a promising candidate for moisture‐responsive actuators.^[^ ^12^, ^29^, ^30^, ^31^, ^32^, ^33^ ^]^ First, GO can be mass‐produced using graphite as raw materials, whose size, defects, and oxidation degree can be well controlled during the chemical exfoliation process.^[^ ^34^, ^35^, ^36^ ^]^ Second, GO can easily be shaped into various morphologies, for instance, continuous films, fibers, and foams, due to their tractable solution‐processing property.^[^ ^37^, ^38^, ^39^ ^]^ Third, the amount and distribution of OCGs on the GO sheets can be flexibly tailored using various chemical/physical treatments.^[^ ^5^, ^40^ ^]^ More importantly, GO has strong interaction with water molecules; the hydroxyl, epoxy, and carboxyl groups on the GO sheets can form hydrogen bonds with the water molecules.^[^ ^41^, ^42^ ^]^ Therefore, GO has excellent water adsorption capability when exposed to moisture, hence it demonstrates a moisture‐responsive swelling effect.^[^ ^43^, ^44^ ^]^ In addition, the diffusion of water molecules among the stacked GO sheets is quick due to the low friction and large slip length.^[^ ^45^ ^]^ All of the above‐mentioned characteristics make GO an ideal moisture responsive smart material for both sensors and actuators.

Herein, we focus on the moisture responsiveness of stacked GO sheets and highlight the recent advances in bioinspired actuators and soft robots based on GO. First, the unique interaction between GO and water molecules is discussed. After that, the bioinspired design principles and working mechanisms of the GO actuators are discussed. Then, the recent progress in GO‐based biomimetic actuators and soft robots that respond to moisture is comprehensively reviewed. Finally, the prospects of this field are briefly discussed.

## 2 Moisture‐Responsive Actuators and Soft Robots Based on GO

Actually, GO has a much longer history than graphene as it was reported as a chemically oxidized graphite, known as graphite oxide, as early as 1859.^[^ ^46^ ^]^ Approximately 100 years later, the method had been improved by Hummers and Offeman in 1957.^[^ ^47^ ^]^ The discovery of graphene triggered the revival of graphite oxide. Nowadays, Hummers’ method has been modified and widely employed for preparing GO for use in developing graphene‐related materials and devices.^[^ ^40^ ^]^ Compared with graphene, GO has a strong interaction with water molecules due to the presence of OCGs, which makes GO a promising candidate for moisture‐responsive smart devices.^[^ ^12^, ^48^, ^49^, ^50^ ^]^ In this section, we mainly discuss the GO–water interaction, design principles, and working mechanisms of the GO‐based actuators, and bioinspired soft robots based on the rational assembly of the GO actuators.

### 2.1 Interaction between GO and Water Molecules

**Figure 1:**
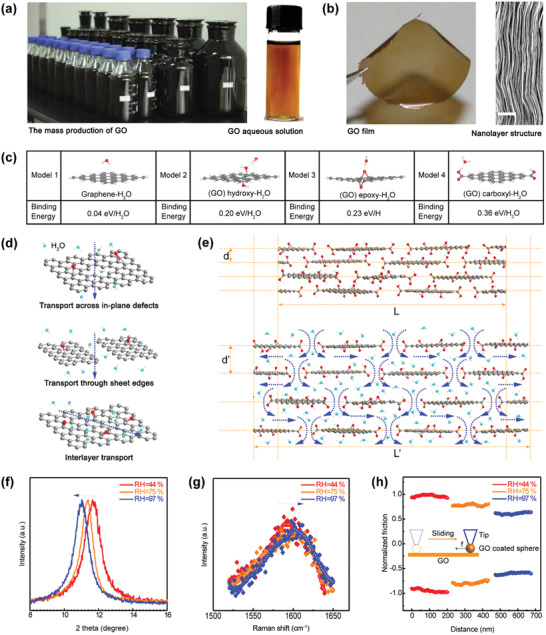
Interaction between GO and water molecules. a) Photograph of the GO solutions. Reproduced under the terms of the CC‐BY Creative Commons Attribution 4.0 International License.^[^ ^51^ ^]^ Copyright 2015, The Authors, Published by Springer Nature. b) Photograph and cross‐sectional SEM image of the GO paper. Reproduced with permission.^[^ ^52^ ^]^ Copyright 2012, American Association for the Advancement of Science. c) The binding energy between a water molecule and graphene as well as GO. d) Schematic view for water transportation path within a GO nanosheet. e) Water permeation through the GO paper. f) X‐ray diffraction patterns, g) Raman spectra, and h) lateral friction force of the GO paper under different RH. c–h) Reproduced with permission.^[^ ^41^ ^]^ Copyright 2019, Wiley‐VCH.

The chemical oxidation of natural graphite imparts abundant hydrophilic OCGs to the graphene sheets, which breaks the *π*‐conjugation.^[^ ^34^ ^]^ As a result, the highly oxidized graphite can be easily exfoliated into a single‐layer GO through ultrasonication, forming a stable GO aqueous solution (**Figure** **1**a).^[^ ^51^ ^]^ Nowadays, continuous efforts are being devoted to GO preparation, hence high‐quality GO aqueous solutions have become commercially available. GO can be considered as OCGs functionalized graphene with abundant defects. The hydrophilic nature endows GO with aqueous solution processing capability, which facilitates the shaping of GO. Soft and robust GO films can be prepared through ordered stacking of individual GO sheets (Figure 1b).^[^ ^52^ ^]^ More importantly, the OCGs show strong interaction with water molecules. According to the first‐principles study, the interaction between pristine graphene region (sp^2^ carbon) and water molecule is through weak van der Waals force, ≈0.04 eV per H_2_O. Nevertheless, in the case of GO sheets, much stronger hydrogen bonds form between OCGs and water molecules. The binding energies for carboxyl, epoxy, and hydroxyl groups are calculated to be 0.36, 0.23, and 0.20 eV per H_2_O, respectively (Figure 1c).^[^ ^41^ ^]^

In addition to the strong GO–water interaction, the ultrafast diffusion rate of water molecules among the GO sheets makes GO sensitive to moisture.^[^ ^45^ ^]^ Geim and co‐workers, reported, for the first time, that the ultrafast water permeation through a stacked GO membrane is about 10^10^ times faster than that of He.^[^ ^52^ ^]^ Generally, the transport of water molecules within GO films occurs through three mechanisms: transport across the in‐plane defects, transport through sheets edges, and interlayer transport (Figure 1d).^[^ ^45^ ^]^ Due to these various mechanisms, GO membranes show relatively high pure water flux $(\prod_{H_{2}O}\;$≈ 10^−5^ mm × g cm^–2^ × s^–1^ × bar^–1^, Figure 1e).^[^ ^52^ ^]^ The ultrafast water transport rate can be attributed to two major points; the abundant oxygen groups that lead to the strong interaction with water molecules and the unique nanolayer structure of GO that can be considered as quantum‐confined superfluidic (QSF) channels for water transportation.^[^ ^53^ ^]^ When actuated by moisture, the GO film adsorbs a large amount of water molecules, leading to its rapid swelling (Figure 1e).^[^ ^41^ ^]^ The adsorption of water molecules leads to structural and property changes in the GO, such as tunable interlayer space,^[^ ^43^, ^54^, ^55^, ^56^ ^]^ interfacial adhesion,^[^ ^57^, ^58^, ^59^ ^]^ and bulk densities.^[^ ^60^ ^]^ To investigate the water adsorption induced swelling effect, X‐ray diffraction (XRD) has been employed to prove the interlayer spacing change in GO under different relative humidity (RH) levels. As shown in Figure 1f, the interlayer spacing of GO can be tuned between 6 and 12 Å, when the RH was varied from 44% to 97%.^[^ ^43^, ^61^ ^]^ The increase in the interlayer spacing under moist conditions confirm the swelling along the normal direction. Nevertheless, a GO film generally has a nanolayer structure due to the stacking of the two‐dimensional (2D) GO sheets. Therefore, the shape change in the GO films along the normal and tangential direction may occur in different ways. Based on the Raman spectra of the GO measured under different RH, the blueshift of the *G* peak can be detected upon water adsorption, which suggests the release of inner stress under high humidity (Figure 1g).^[^ ^41^ ^]^ Therefore, the swelling along the tangential direction can be attributed to the sliding of the GO sheets. The sliding friction test carried out by atomic force microscopy (AFM) further supports this hypothesis (Figure 1h). When the RH increases from 44% to 97%, the friction force between the GO nanosheets on the AFM tip and that on the substrate decreased by ≈40% due to the presence of a lubricating water layer under high moisture conditions,^[^ ^41^ ^]^ which facilitates the sliding of the GO sheets. Based on these experimental results, the moisture triggered swelling behavior of GO is easily understood. Meanwhile, the adsorption/desorption of water gives rise to the expansion/contraction of the GO film along both the normal direction with respect to the film thickness and the tangential direction with respect to the size of the film. Due to the ultrafast transport of water molecules, GO demonstrates a fast and dynamic response to environmental humidity. As compared with other moisture‐responsive materials, for instance, hydrogels, GO possesses several distinct advantages. First, GO has high oxygen content (e.g., the C/O atom ratio < 3), which determines the strong interaction with water molecules.^[^ ^42^, ^62^ ^]^ In addition, GO has a unique layered nanostructure with the interlayer spacing between 6 and 12 Å, which can be considered as natural quantum‐confined superfluidic channels for ultrafast water transportation.^[^ ^43^, ^61^ ^]^ More importantly, the type, amount, and distribution of the OCGs on the GO sheets can be tuned using appropriate chemical/physical treatments.^[^ ^31^, ^63^, ^64^, ^65^ ^]^ This provides an opportunity to control the deformation of GO the sheets when used as actuators.

### 2.2 Design Principles and Working Mechanisms of GO‐Based Actuators

**Figure 2:**
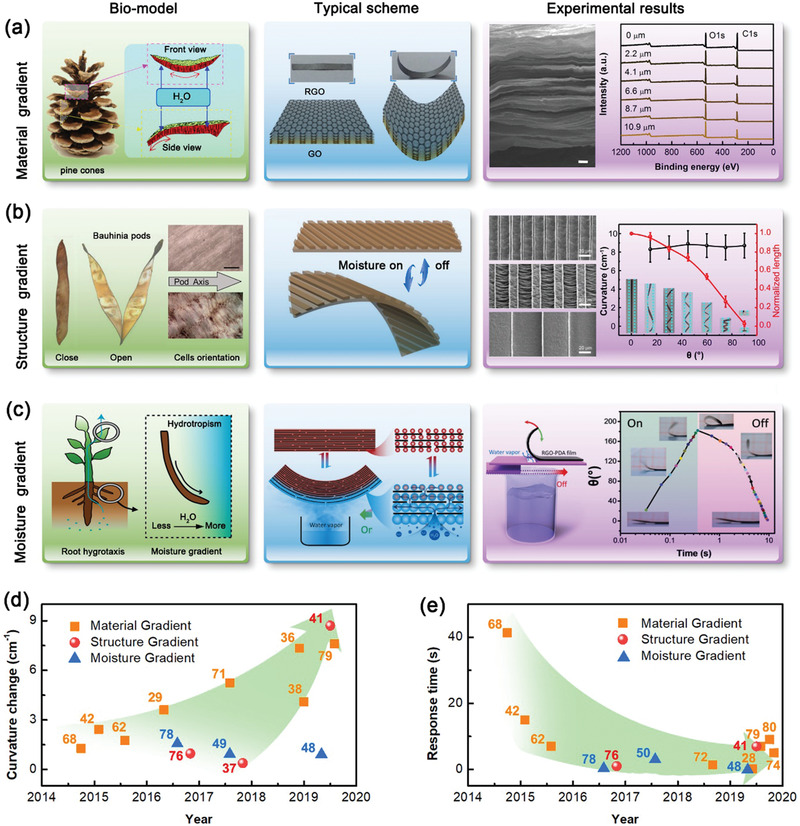
Design principles and working mechanisms of the GO‐based actuators. Scheme of the bio‐model, typical scheme, and experimental results of moisture‐responsive GO‐based actuators based on a) material gradient, b) structure gradient, and c) moisture gradient. a, left image) For the pine cone image: Reproduced under the terms of the CC‐BY Creative Commons Attribution 4.0 International License.^[^ ^66^ ^]^ Copyright 2018, The Authors, Published by Elsevier. a, middle and right images) Reproduced with permission.^[^ ^62^ ^]^ Copyright 2015, Wiley‐VCH. b, left image) For the *Bauhinia* pods image: Reproduced with permission.^[^ ^99^ ^]^ Copyright 2011, American Association for the Advancement of Science. b, middle and right images) Reproduced with permission.^[^ ^41^ ^]^ Copyright 2019, Wiley‐VCH. c, left image) For the root hygrotaxis image: Reproduced with permission.^[^ ^77^ ^]^ Copyright 2019, The Botanical Society of Japan and Springer Japan KK, part of Springer Nature. c, middle and right images) Reproduced with permission.^[^ ^78^ ^]^ Copyright 2016, Wiley‐VCH. The excellent actuation performance: d) the curvature change and e) response time among the GO‐based actuators reported within the last 5 years.

The sensitive dimension change with environmental humidity makes GO a smart material for use in moisture‐responsive actuators. However, the moisture‐triggered expansion or contraction of a GO film is usually isotropic along the normal direction, which cannot cause any apparent deformation. Therefore, divers design principles have been proposed to fabricate asymmetric GO structures, which can exhibit apparent deformation, for use in moisture‐responsive actuators. To reach this end, natural organisms inspire scientists globally. By mimicking the working principle of biological systems, several moisture‐responsive actuators have been successfully developed based on the GO films (**Figure** **2**). Typically, controllable deformation can be achieved by forming material gradient, structure gradient, or moisture gradient.

#### 2.2.1 Material Gradient

In nature, moisture can trigger inhomogeneous local swelling/shrinking of pinecones due to the presence of two kinds of tissues at the bottom of each scale (Figure 2a, left).^[^ ^66^ ^]^ This interesting natural phenomenon inspired the development of moisture‐responsive actuators by tuning the material gradient. Combining a moisture‐responsive material with a moisture‐inert material can directly form a material gradient. Accordingly, various bi‐/multi‐layer structures that consist of two or more materials of distinct water adsorption/desorption properties have been prepared to perform controllable moisture responsive bending deformations. For instance, Ruoff and co‐workers GO/multi‐walled carbon nanotubes (GO/MWCNTs) bilayer papers for moisture actuation.^[^ ^67^ ^]^ When the RH increases, the GO layer expands upon water adsorption, whereas the MWCNT layer remains inert. Consequently, the bilayer paper deforms towards the MWCNT side due to the interfacial strain mismatch. Following this design principle, several approaches, including vacuum filtration, blade coating, and casting, have been employed to prepare moisture responsive bimorph actuators using GO and various inert materials (e.g., PDA‐RGO/NOA‐63,^[^ ^68^ ^]^ GO/RGO,^[^ ^69^ ^]^ GO/polyvinylidene fluoride,^[^ ^70^ ^]^ GO/polypropylene,^[^ ^71^ ^]^ and GO/CNT‐polydimethylsiloxane^[^ ^72^ ^]^).

Generally, the interaction between GO and other functional materials is through relatively weak van der Waals force. Thus, bimorph actuators prepared from moisture‐active GO and moisture‐inert materials may suffer from serious problems due to the interlayer adhesion, especially during frequent bending and straightening. To address this issue, various chemical/physical modification strategies have been developed to tailor the material property gradient of GO by selective removal of OCGs.^[^ ^42^, ^62^, ^65^ ^]^ For example, Qu and co‐workers reported laser selective reduction of GO fibers during the development of graphene and GO (G/GO) fiber actuators, in which sophisticated shape changes, such as hook, S‐shaped actuation, and a folded structure, were achieved.^[^ ^65^ ^]^ In our work, we reported the self‐controlled photoreduction of GO for developing moisture‐responsive GO papers for robotic design.^[^ ^42^, ^62^ ^]^ The gradient of the OCGs formed naturally along the lateral direction, because of the limited light transmittance. The thermal relaxation can be suppressed during the photoreduction of thick GO papers (Figure 2a, middle). The as‐formed OCG gradient, which gives rise to the asymmetric water adsorption under moisture actuation, has been confirmed experimentally (Figure 2a, right). In addition to the gradient control, the photoreduction treatment permits flexible patterning, which makes it possible to perform more complex actions, such as twisting. Besides, GO actuators with a material gradient have been fabricated by gradient assembly of the GO and other guest nanomaterials (e.g., magnetic nanoparticles, and polymer nanospheres).^[^ ^73^, ^74^ ^]^ Instead of forming a bilayer structure, the asymmetric distribution of the guest nanomaterials also leads to moisture responsive deformation. Moreover, due to the absence of sharp interface interfaces across the materials, delamination during frequent actuation can be avoided, leading to improved stability.

#### 2.2.2 Structure Gradient

In addition to the material gradient, the creation of a structure gradient also enables moisture actuation of the GO. This concept is inspired by the opening of the chiral seed pods (*Bauhinia*), which forms as structure gradient through cell arrangement and orientation and enables moisture induced flat‐to‐helical transition during pod opening (Figure 2b, left).^[^ ^75^ ^]^ By making full use of the structure gradient, moisture‐responsive GO actuators have also been fabricated via asymmetrical structuring. For instance, Qu and co‐workers reported the self‐assembly of GO sheets into an asymmetric film, demonstrating reversible deformation in response to moisture.^[^ ^37^, ^76^ ^]^ As compared to the GO‐based bimorph actuators, the structure gradient‐enabled GO actuators may have improved durability due to the absence of the material interface and significantly improved response time because of the asymmetric micro‐nanostructures.

Recently, our group created a structure gradient on a GO paper by soft lithography (Figure 2b, middle).^[^ ^41^ ^]^ The unilateral microstructures can act as quantum‐confined superfluidic channels and facilitate the adsorption, transport, and desorption of water. As a result, unbalanced stress can be generated that can induce predictable deformation of the film. Under moisture actuation, the asymmetrical GO film bends against the grating direction towards the flat side. Accordingly, the deformation behavior can be tuned by controlling the orientation of the gratings, enabling more complex deformations such as bending, helical bending, and chiral bending (Figure 2b, right). More importantly, this strategy is scalable, it permits large‐area preparation, and can be applied to other 2D materials, such as MXene.

#### 2.2.3 Moisture Gradient

Inspired by the hygrotaxis of the roots, manipulation of the GO actuators can also be realized by controlling the environmental moisture gradient (Figure 2c, left).^[^ ^77^ ^]^ For example, Chen and co‐workers prepared reduced graphene oxide‐polydopamine (RGO‐PDA) films, and demonstrated controllable actuation using the water molecules gradient (Figure 2c, middle).^[^ ^78^ ^]^ Because of the asymmetric moisture field, the side directly exposed to the moisture absorbed more water molecules than the other side, leading to fast and large‐scale bending of the film. By taking advantage of the sensitive response to the moisture gradient (Figure 2c, right), such GO‐based actuators can be used as smart valves of a sealing film for controllable sealing or release of vapor inside a container. For example, in a similar work reported by Wang et al., a moisture/alkane dual‐responsive actuator was fabricated based on the GO and polydimethylsiloxane bilayer structure.^[^ ^79^ ^]^ A smart gas valve was demonstrated for selective gas transportation.

Compared to the material/structure gradient that can be tailored in a controlled manner, it is almost impossible to precisely control the moisture gradient in the environment. Therefore, in most cases, the moisture gradient is a complementary strategy for moisture actuation. Figure 2d,e shows the excellent actuation performance of GO‐based actuators reported within the last 5 years. Most of the reported works are designed based on the materials gradient. The performance of the GO‐based actuators represented by the deformation degree (e.g., curvature for bending) and response time has been improved significantly. Particularly, the actuators based on the structure gradient had large deformation curvatures and short response times. In addition, the presence of a moisture gradient can further improve the responsiveness of the actuators.

### 2.3 Bioinspired Soft Robots

**Figure 3:**
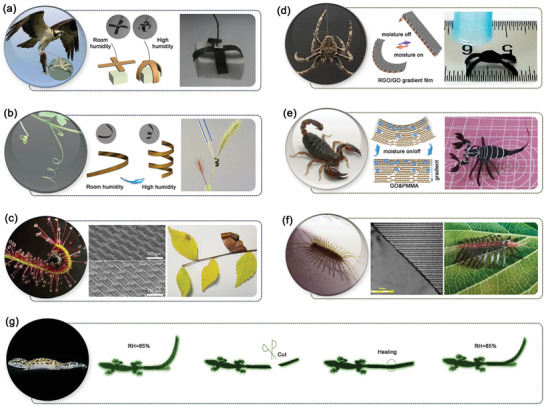
Bioinspired soft robots. a) Gripping. Reproduced under the terms of the CC‐BY Creative Commons Attribution 4.0 International License.^[^ ^80^ ^]^ Copyright 2019, The Authors, Published by Springer Nature. b) Curling. Reproduced with permission.^[^ ^62^ ^]^ Copyright 2015, Wiley‐VCH. c) Rolling. Reproduced with permission.^[^ ^41^ ^]^ Copyright 2019, Wiley‐VCH. d) Bending. For the spider image: Reproduced under the terms of the CC‐BY Creative Commons Attribution 4.0 International License.^[^ ^100^ ^]^ Copyright 2019, The Authors, Published by Springer Nature. For the scheme and the bending image: Reproduced with permission.^[^ ^81^ ^]^ Copyright 2017, Optical Society of America. e) Crawling. For the scorpion image: Reproduced with permission.^[^ ^101^ ^]^ Copyright 2019, Elsevier. For the scheme and the scorpion robot image: Reproduced with permission.^[^ ^74^ ^]^ Copyright 2019, American Chemical Society. f) Twisting. Reproduced with permission.^[^ ^41^ ^]^ Copyright 2019, Wiley‐VCH. g) Self‐healing. For the gecko image: Reproduced with permission.^[^ ^102^ ^]^ Copyright 2014, Wiley‐VCH. For the GO self‐healing tail images: Reproduced with permission.^[^ ^37^ ^]^ Copyright 2019, Wiley‐VCH.

Nature has been considered a perpetual source of inspiration for innovative designs of artificial automated devices and systems. Using the moisture‐responsive actuators, GO‐based soft robots were also developed by mimicking the behavior of natural organisms (**Figure** **3**). For instance, inspired by eagle claws,^[^ ^80^ ^]^ a smart grasper with stimuli‐response arms, which can catch object upon exposure to moisture and release it in dry air, has been fabricated using GO/polypyrrole actuators as smart “fingers” (Figure 3a). The gripping ability can be attributed to the bending deformation of the GO actuators. To get better control over the gripping performance, the GO actuators have been integrated with a plastic tube that connects to a moisture source.^[^ ^42^ ^]^ In this way, the capture and release functions can be triggered in a controlled manner. In addition to simple gripping, inspired by the climbing behavior of plant tendrils,^[^ ^62^ ^]^ GO stripes that exhibit curling deformation have been prepared by simultaneously tailoring the OCG gradient and RGO patterns (Figure 3b). The curling ribbon can deform into a helical structure and act as a manipulator for controllable objects transport under moisture actuation. Recently, structure gradient‐enabled GO actuators demonstrated a much faster response to moisture, which significantly improves the capturing ability of the actuators. Similar to the *Drosera*, the leaf‐shape GO actuator is capable of catching ladybugs when actuated by moisture (Figure 3c).^[^ ^41^ ^]^ The above‐mentioned works highlight the rational design and development of smart gripping robots using GO actuators.

In addition to grapping, motion is another important element for robots. The moisture‐induced bending behavior makes GO actuators promising candidates for designing crawler soft robots.^[^ ^41^, ^65^, ^74^, ^81^, ^82^ ^]^ In a pioneering work, Qu and co‐workers, successfully demonstrated the actuation of a fiber walker (a selectively reduced GO fiber) that moved between two glass slide. The moisture‐induced bending deformation was responsible for the walking behavior.^[^ ^65^ ^]^ Subsequently, various walking robots with biomimetic shapes have been developed to achieve crawling. These simple yet powerful robots have a thin film structure, similar to a piece of paper. Under intermittent moisture actuation, they can crawl on a rough surface without an external power source (Figure 3d).^[^ ^81^ ^]^ Besides, the GO actuators can be assembled to form more complex soft robots. In a recently published paper, a dual‐responsive scorpion robot, with claws, legs, and a long tail, was fabricated based on the GO actuators (Figure 3e).^[^ ^74^ ^]^ Under moisture actuation, the scorpion robot sprawled and in response to light irradiation, it stood up. At the same time, the claws closed, and the tail curled up. Nevertheless, the simple bending of robotic “legs” usually causes lateral movement, whereas real insects generally crawl forward. To address this issue, our group fabricated a centipede‐like soft walking robot with ten pairs of “smart legs” that enable chiral twisting (Figure 3f).^[^ ^41^ ^]^ Due to the presence of the grating structures with proper orientation, the centipede robot crawls forward through the cooperation of the twisting legs. In this way, it can walk along a straight line at a uniform speed.

The performance of the GO soft robots is not limited to gripping and motion. By mimicking natural organisms, various robotic devices can be developed by rational design and assembly of the GO actuators.^[^ ^44^, ^62^ ^]^ For example, inspired by the respiratory cilia that can sweep tiny particles away to prevent bacterial accumulation, our group manufactured an artificial cilia array by assembling two kinds of GO actuators to realize a sweeping performance.^[^ ^62^ ^]^ Additionally, to realize more complex deformation, our group recently demonstrated the programmable deformation of a patterned GO actuator swarm.^[^ ^44^ ^]^ The idea was inspired by the collective coupling and coordination of living cells. By programming the geometries and orientations of the SU‐8 patterns underneath a GO film, a swarm of GO actuators was integrated within one film, enabling predictable and complex deformations, such as bending, twisting, coiling, asymmetric bending, and three‐dimensional (3D) folding. Therefore, GO possesses unique features that are of benefit to actuator design, for instance, the mechanical strength and self‐healing properties. Inspired by the lizard tail, Qu and co‐workers developed a self‐healing walker robot using GO.^[^ ^37^ ^]^ The GO tail of the robot healed more than ten times, and the motion capacity partly recovered (Figure 3g).

## 3 Conclusions and Outlook

In summary, we provided in this article an overview of bio‐inspired actuators and soft robots based on the moisture‐responsive property of GO. With abundant OCGs on GO sheets, the as‐formed GO films, fibers, and foams show strong interaction with water. According to the theoretical study and experimental results, water molecules interact with GO by forming hydrogen bonds. The quasi‐ordered nanolayer structure (interlayer spacing, 6–12 Å) can be considered as a QSF system, and the permeation of water through a GO membrane is ultrafast, about 10^10^ times faster than He. The unique interaction with water endows the GO with sensitive moisture responsive properties, enabling moisture actuation.

To get better control of the deformation of the GO, three actuation strategies were proposed that were inspired by nature. First, the GO actuators were fabricated based on the material gradient. The combination of the moisture‐responsive GO with another moisture‐inert material layer is a simple yet effective way to achieve a material gradient. However, the interlayer adhesion at the bilayer interface would be a potential issue that restricts long‐term durability. As an alternative, controllable modification of the GO along the normal direction or gradient assembly of the other guest nanomaterials within the GO would avoid the presence of the bilayer interface, which consequently promotes the stability during frequent deformation. Second, a structure gradient can also be created along the normal direction of a GO film for controllable moisture actuation. By tailoring the QSF channels of the GO, the resultant GO actuators can achieve a much faster response to moisture and more complex deformation. Additionally, the moisture gradient in the environment would also contribute to the actuation of the GO. Nevertheless, it is almost impossible to control the spatial moisture field precisely, thus the moisture gradient can be employed as a complementary mechanism for moisture actuation.

Recently, inspired by the behavior of various natural organisms, GO‐based soft robots capable of grapping and crawling have been successfully developed by rational assembly of the GO actuators. The predictable deformation capability, dynamic response to moisture, and biomimetic design principle make the GO‐based soft robots promising for various cutting‐edge applications, including e‐skin, MEMS, and lab‐on‐a‐chip systems. Notably, graphene offers outstanding flexibility, mechanical strength, and electric properties, and thus contributes greatly to flexible electronics.^[^ ^40^ ^]^ Particularly, the GO, which allows flexible patterning, structuring, and controllable tuning of its physical/chemical properties, has emerged as an important raw material for developing flexible electronic devices, such as sensors, optoelectronic devices, and energy storage devices. Considering the simple planar device structure, the flexible electronic devices can be integrated with GO‐based soft robots, which would undoubtedly promote their comprehensive abilities for practical usage. Taking the smart gripper as an example, the integration of a GO‐based pressure sensor at the tip of the gripper would make it smarter; and the combination of energy generation/storage devices with the gripper would achieve self‐powering. In this regard, the GO‐based actuators and robots may have great potential for use in the development of flexible MEMS that can meet some special requirements in the future.

Nevertheless, despite the rapid advances, the GO‐based actuators and soft robots are still at an early stage. There exist several challenges for further advancement. For instance, the currently reported GO actuators are mainly based on 2D films. Configuration variation from one‐dimensional fiber to complex 3D structures may be required for actuator design. However, the processing of the GO is compatible with various functional nanomaterials that can be dispersed in aqueous solutions. Thus, the gradient assembly of the GO and other nanomaterials into asymmetric hybrids may provide more possibilities for developing soft robots with special functionalities or multi‐signal responsiveness. More importantly, as a proof‐of‐concept, the full potential of moisture responsive soft robots has been demonstrated. However, practical applications of such smart and automated devices are still rare. In addition to the moisture responsiveness, the GO also shows many unique properties, for instance, excellent mechanical strength, tunable physical/chemical properties, and biocompatibility. Therefore, the GO‐based actuators and soft robots may exhibit distinct superiority over hydrogel‐based actuators and soft robots. For example, considering the good biocompatibility of graphene, the biological properties of GO have been well studied over the past decade. These reveal the great potential for use in bacteria killing,^[^ ^83^, ^84^, ^85^, ^86^ ^]^ drug delivery,^[^ ^87^, ^88^, ^89^ ^]^ biosensing,^[^ ^90^, ^91^, ^92^ ^]^ and tissue engineering.^[^ ^93^, ^94^, ^95^ ^]^ Therefore, the smart GO grippers that have antibacterial properties can be employed as a mechanical manipulator for minimally invasive surgery or organ‐on‐a‐chip systems. For drug delivery applications, the GO can achieve strong interaction with anti‐cancer drugs and shows sensitive response to pH value, thermal and photothermal signals, enabling large amount drug loading, controllable releasing, target delivery, and biodegradation. Besides, GO and its hybrids have been widely used as scaffolds for culturing human mesenchymal stem cells;^[^ ^96^, ^97^, ^98^ ^]^ the GO‐based robotic structures may find practical applications in tissue engineering. Soon, triggered by the sensitive moisture responsiveness of the GO actuators and its potential importance to soft robotics, it is expected that increased research efforts would be devoted to this field, leading to breakthroughs in both fundamental science and practical applications.

## Conflict of Interest

The authors declare no conflict of interest.
